# Chlorotoxin: A Helpful Natural Scorpion Peptide to Diagnose Glioma and Fight Tumor Invasion

**DOI:** 10.3390/toxins7041079

**Published:** 2015-03-27

**Authors:** Lucie Dardevet, Dipti Rani, Tarek Abd El Aziz, Ingrid Bazin, Jean-Marc Sabatier, Mahmoud Fadl, Elisabeth Brambilla, Michel De Waard

**Affiliations:** 1 Grenoble Neuroscience Institute, Inserm U836, Team 3, Chemin Fortuné Ferrini, Bâtiment Edmond Safra, 38042 Grenoble Cedex 09, France; E-Mails: lucie.dardevet@gmail.com (L.D.); diptidahiya@gmail.com (D.R.); tarek.mohamed@mu.edu.eg (T.A.E.A.); 2 Science Technology Health, Université Joseph Fourier, BP53, 38041 Grenoble, France; E-Mail: EBrambilla@chu-grenoble.fr; 3 Labex Ion Channel Science and Therapeutics, 660 route des lucioles, 06560 Valbonne, France; 4 Zoology Department, Faculty of Science, Minia University, 61519 Minia, Egypt; E-Mail: mahmoudfs2000@yahoo.com; 5 Ecole des Mines d’Ales, 6 av de Clavieres, 30100 Ales Cedex, France; E-Mail: ingrid.bazin@mines-ales.fr; 6 Inserm UMR 1097, 163, Avenue de Luminy, 13288 Marseille Cedex 09, France; E-Mail: sabatier.jm1@libertysurf.fr; 7 Institut Albert Bonniot, Inserm U823, Rond-Point de la Chantourne, 38706 La Tronche Cedex, France; 8 Smartox Biotechnology, 570 Rue de la Chimie, Bâtiment Nanobio campus, 38400 Saint-Martin d’Hères, France

**Keywords:** chlorotoxin, glioma, cancer, targeting, diagnosis, treatment, therapy, chloride channel, Annexin A2, metalloprotease

## Abstract

Chlorotoxin is a small 36 amino-acid peptide identified from the venom of the scorpion *Leiurus quinquestriatus*. Initially, chlorotoxin was used as a pharmacological tool to characterize chloride channels. While studying glioma-specific chloride currents, it was soon discovered that chlorotoxin possesses targeting properties towards cancer cells including glioma, melanoma, small cell lung carcinoma, neuroblastoma and medulloblastoma. The investigation of the mechanism of action of chlorotoxin has been challenging because its cell surface receptor target remains under questioning since two other receptors have been claimed besides chloride channels. Efforts on chlorotoxin-based applications focused on producing analogues helpful for glioma diagnosis, imaging and treatment. These efforts are welcome since gliomas are very aggressive brain cancers, close to impossible to cure with the current therapeutic arsenal. Among all the chlorotoxin-based strategies, the most promising one to enhance patient mean survival time appears to be the use of chlorotoxin as a targeting agent for the delivery of anti-tumor agents. Finally, the discovery of chlorotoxin has led to the screening of other scorpion venoms to identify chlorotoxin-like peptides. So far several new candidates have been identified. Only detailed research and clinical investigations will tell us if they share the same anti-tumor potential as chlorotoxin.

## 1. Glioma, a Difficult to Cure Human Brain Cancer

Amongst primary brain tumors, gliomas can be considered as the most lethal malignant tumors. This is a family of central nervous system (CNS) tumors derived from differentiated glial cells or glioblastoma stem-like cells [1,2]. It is composed of glioblastoma multiforme (GBM), anaplastic astrocytoma, astrocytoma and oligodendroglioma. The two first gliomas occur at an incidence of 78% of all the primary brain tumors. Gliomas represent very aggressive brain cancers characterized with a fast cell proliferation rate and a strong tendency to invade healthy brain tissue (French Foundation for medical research). Even low-grade gliomas infiltrate the entire brain. The molecular mechanisms of brain tumor invasion are complex. They involve (i) modification of receptor-mediated adhesive properties of tumors cells; (ii) degradation and remodeling of the extracellular matrix by tumor-secreted metalloproteinases; and (iii) creation of an intercellular space for tumor cell invasion (See Box 1). Standard treatment involves surgery whenever the tumor mass is accessible, followed by chemoradiation and adjuvant chemotherapy with temozolomide. In spite of this therapeutic arsenal, the survival rate of patients rarely exceeds sixteen months [3]. At best, 3% of the patients may benefit of a five-year survival time. This fatal outcome points to other major issues with gliomas, which is their resistance to radiation and chemotherapy, and the difficulty to accurately localize them within the tissue. Although it is possible to roughly visualize the tumor with current imaging techniques, it is very tedious to determine the exact boundaries of tumor invasion. In addition, diagnosis of this cancer still requires tissue biopsy and histopathological analyses. Histological features of interest comprise vascular proliferation and focal necrosis.

Box 1 **Mechanism of glioma cell invasion.**

Cell invasion is a natural mechanism that plays an important role in embryonic development, wound healing, immune response and tissue repair. In this situation, the cell migrates on the influence of chemical signals, physical cues and physicochemical processes. Unfortunately, when this complex mechanism is affected by deleterious mutations, an uncontrolled cell invasion leads to the development of several pathologies (e.g., arthritis, atherosclerosis, aneurism, chronic obstructive pulmonary disease, etc.). In the case of cancer, it leads to metastasis or an infiltrative tumor [4]. One of the major characteristics of glioma cells is their propensity to invade healthy brain tissue. The principal mode of invasion of a glioma cell is a single cell invasion, which can be decomposed into five steps: (i) change in glioma cell morphology (formation of membrane protrusions); (ii) interaction between membrane protrusions and extracellular matrix (ECM) to obtain traction; (iii) degradation of ECM by matrix metalloprotease (MMP)-proteins among others; (iv) change of shape (contraction) for the cell to cross the “ECM hole”; (v) detachment of the rear end connection (the cell moves forward). The key abilities for glioma cells to invade healthy brain tissue are modification of cell adhesion property, degradation of ECM, and change of shape. The invading tumor cells do not spread anarchically in the brain, the degradation of ECM occurs at the border between the tumor and the healthy tissue [5]. The invading cells spread following existing anatomical structures such as nerves and blood vessels [6]. During the first steps of invasion, glioma cells will interact with ECM and its environment thanks to adhesion proteins, especially integrins, giving the cell traction points to displace. Then, using proteolytic enzymes, such as the MMP proteins, the cells begin to degrade the ECM, to create a space in which through which they can pass. In order to move through the newly created space, glioma cells need a change in shape and volume. At this point, glioma cells use ionic channels (Cl^−^ and K^+^ channel) to shrink, and so fit the space to pursue the invasion. Because of adhesion molecules and specific cell surface receptors, cancer cells move forward in the invasive direction [4,6,7]. When the invasive cells reach a certain distance from the primary tumor mass, they re-enter the cell cycle and form a new tumor mass [8].

In this context, therefore, the identification of marker molecules, specifically binding to tumor cells, would represent a tremendous asset to researchers and clinicians aiming at precisely localizing the tumor mass. If, in addition, such a marker molecule could selectively deliver therapeutic agents to these cancer cells, this would enlarge the arsenal of chemical entities used in therapeutics to treat gliomas. Tumor-specific targeted therapies are increasingly used strategies that have demonstrated their potential through the emergence and development of antibodies, antibody-like ligands, proteins, peptides or chemical drugs to identify, localize or treat cancers [9]. The principle of targeted therapies is based on the identification of a suitable molecular target expressed at the surface of a given cell type. Most of the time, it is a membrane receptor that is over-expressed or preferentially expressed in cancer cells. Targeting the cancer cells ensures that the normal brain tissue is not affected by a cytotoxic drug that would be conjugated to the ligand that binds to the specific cell target. All of the targeting agents should have tolerable cell toxicities, fit mass production criteria, and have a high specificity or selectivity of binding to tumors cell or other tumor-related targets (vascular cells). For gliomas, in addition to these characteristics, the ability of the targeting agent to naturally cross the blood brain barrier (BBB) would be a desirable property. Alternatively, this targeting agent should at least cross the blood-brain tumor barrier (BBTB). This would prevent the need for a loco-regional injection to deliver the targeting agent to the tumor site within the brain. In spite of these evident advantages, investigators were unable to unequivocally identify glioma-specific markers so far. Reasons for this problematic deficit come from the great genetic and antigenic variability of gliomas. This further explains why the diagnosis of this cancer type still requires tissue biopsy and histopathological analyses. This situation has recently changed with the identification of chlorotoxin (CTX) for glioma detection.

## 2. Animal Toxins, Wonderful Potent Natural Peptides for Therapy and Diagnosis

Peptides are increasingly considered as good drug candidates for therapeutic applications. In 2009, 438 peptides were considered by the pharmaceutical industry in their development programs. Of these candidates, 72 were in Phase III clinical trials. Forty-eight peptides are now on the market. In 2007, four of them reached global sales over 500 million dollars each: copaxane ($3.33 billions), lupron ($1.88 billions), byetta ($967 millions) and forteo ($709 millions). The majority of these peptides target G protein coupled receptors, although other targets are increasingly common, such as ion channels.

A complete report on the development of peptides as therapeutic drugs can be requested from http://www.peptidetherapeutics.org. Obviously, it may seem odd at first glance to consider animal toxins as potential drugs. However, animal venoms are enriched sources of biologically active peptides of about 100 to a 1000 different components. In addition, peptides issued from venoms are tailored by Nature to be extremely stable *in vivo*. Different from synthetic chemical libraries, all toxins present in venoms are active, often at nanomolar affinities. In addition, while venoms can be toxic, the toxicity is mainly due to a few peptide members or to the synergistic effect of a combination of peptides. As a matter of fact, the vast majority of venom components possesses interesting therapeutic potential that can be usefully exploited. Hence, several toxins are actually in various clinical phases for the treatment of pain, epilepsy, cancer, atherosclerosis and cardiac failure. It might be of interest that many of these natural peptides target ion channels, ionotropic receptors, transporters and G protein coupled receptors. They also have been found to target enzymes, all constituting major pharmacological classes for the treatment of pathological conditions. Other unusual cell targets have been reported. Disintegrins, a group of snake venom toxins, have the potential to block cancer cell migration and invasion *via* an RGD-dependent sequence that interacts with integrins, a class of membrane proteins required for cell immobilization through interaction with the extracellular matrix [10,11].

## 3. Chlorotoxin, a Natural Peptide Acting as a Potent Glioma Marker

**Figure 1:**
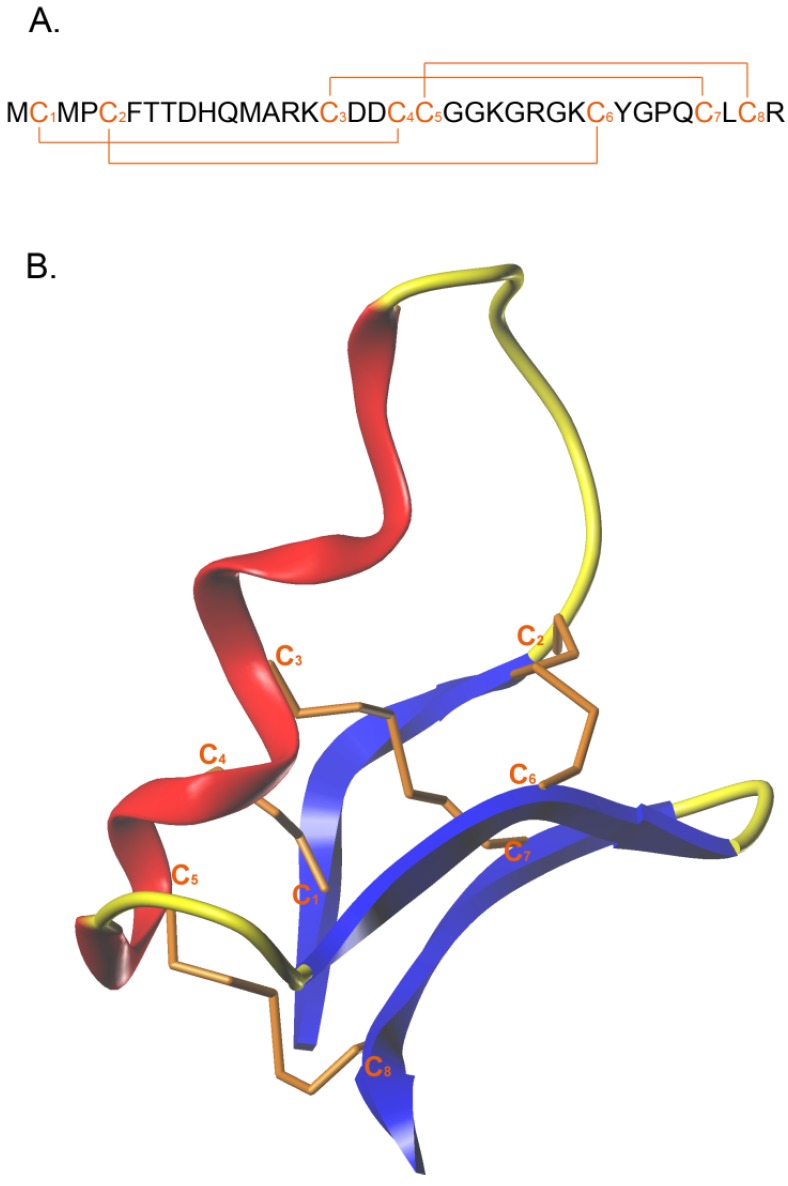
Amino acid sequence and 3D representation of CTX: (**A**) Amino acid sequence of CTX with the eight cysteine residues and the four disulfide bridge in orange; (**B**) 3D structure of CTX, obtain from 1CHL PDB file; α-helix in red, β sheet in blue and disulfide bridge in orange.

CTX is a small neurotoxin of 36 amino acids, isolated in 1993 from the venom of the Israeli scorpion *Leiurus quinquestriatus* [12]. It holds great promise for the treatment of glioma and other solid tumors. CTX has a compact structure, which is maintained by four disulfide bonds that connect the eight cysteine residues present in the sequence. The amino acid sequence of this natural peptide is detailed in Figure 1A. The cysteine pattern adopted is of the type C_1_–C_4_, C_2_–C_6_, C_3_–C_7_ and C_5_–C_8_. Three small antiparallel β-sheets are packed against an α-helix [12] (Figure 1B). With its compact structure, CTX was proposed to cross the BBB (TransMolecular, Inc., Cambridge, MA, USA; unpublished data). However, the data were not sufficiently substantiated to firmly demonstrate that CTX crosses the BBB rather than the BBTB. Nevertheless, it was clear that CTX diffused deeply into the tumors while other targeting agents such as antibodies could not [9,13]. Another report showed that in transgenic mice that spontaneously develop brain medulloblastoma cancers, a fluorescently-tagged Cy5.5-CTX labeled cancer cells while no disruption of the BBB was observed (exclusion of blue Evans labeling of brain structures) [14]. Since this is the only study that investigates the issue of the BBB crossing by CTX and that BBB disturbance by tumors may depend on the tumor type and the stage of progression, it remains cautious to state that CTX crosses at least the BBTB. As a component of the scorpion venom, CTX induces paralysis in small insects or other invertebrates that may be stung by the scorpion. When injected in vertebrates, however, no apparent signs of toxicity have been observed. This indicates that the binding of CTX on its cell surface receptor has no cell toxic or unwanted physiological consequences, as observed for many other animal toxins.

As developed in Section 4, none of the proposed receptors of CTX present important properties for cell survival, although they can be considered as pro-factors for glioma development.

The amino acid sequence of CTX presents several interesting features for its labeling by a number of compounds. Following chemical modification, CTX can then be used to (i) identify its receptor; (ii) characterize its pharmacological properties; and (iii) investigate its mode of action. Several types of chemical modifications have been performed. CTX contains a single tyrosine residue at position 29 that can be used successfully for iodination. ^125^I-CTX has been used to determine the number of receptor binding sites and the affinity of CTX for these sites from cultured glioma cell lines [15]. ^131^I-CTX was used instead of ^125^I-CTX for *in vivo* approaches to obtain gamma-ray scintigram scans because of its higher γ emission properties. Intact activity after iodination of Tyr^29^ demonstrates that this amino acid is not critical for CTX activity. Lysine residues can also be used to easily perform conjugation of active substances thanks to a wide range of cross-linking reagents. Finally, Oregon green-labeled CTX and a complex of biotin-CTX/avidin-rhodamine have been used for immunohistochemical detection of glioma cells in culture, human glioma xenografts in SCID mice or in patients biopsies [15]. For the biopsies, the intensity of the labeling was found to increase with the malignancy grade of the tumors.

**Table 1:** Summary of various human tissues stained with CTX.

Tissues origin	Tissues types	Cases	Results
***Primary brain tumors (glioma)***	Glioblastoma multiforme WHO Grade IV	31	31 positive
	Anaplastic astrocytoma WHO Grade III	7	7 positive
	Low-grade astrocytoma WHO Grade II	4	4 positive
	Pilocytic astrocytoma WHO Grade I	14	13 positive, 1 negative
	Other ungraded gliomas	5	4 positive, 1 negative
	Oligodendroglioma	8	8 positive
	Gliosarcoma	2	2 positive
	Ganglioglioma	5	5 positive
	Meningioma	25	20 positive, 5 negative
	Ependymona	3	3 positive
***Other normal or diseased brain tissue***	Alzheimer’s brain	8	8 negative
	Parkinson’s/schizophrenic brain	4	4 negative (2 each)
	Normal brain or uninvolved tissue of brain cancer patients	29	21 negative, 8 positive *
	Epilepsy/gliosis/stroke brain	6	6 negative ^‡^
***Neuroectodermal tumors***	Medulloblastoma	4	4 positive
	Neuroblastoma	9	8 positive 1 negative
	Ganglioneuroma	4	4 positive
	Melanoma (metastatic)	11	11 positive
	Melanoma (primary)	5	5 positive
	Pheochromocytoma	6	5 positive, 1 negative
	Ewing’s sarcoma	2	2 positive
	Primitive neuroectodermal tumors	2	2 negative
	Small cell lung carcinoma	6	5 positive, 1 negative
	Schwannoma	4	4 positive
***Other brain tumors***	Epidermoid cysts	5	1 positive, 4 negative
	Brain tumors of unknown pathology	9	9 positive
	Pituitary gland of glioblastoma multiforme pt.	2	2 positive
	Metastatic tumors to brain	17	15 positive, 2 negative ^§^
***Other tumors***	Breast cancer	14	13 positive, 1 negative
	Breast cancer metastases	11	11 positive
	Kidney cancer	3	3 positive
	Liver cancer	3	3 positive
	Lung cancer	3	3 positive
	Lymphoma	2	2 positive
	Ovarian cancer	3	3 positive
	Pancreatic cancer	3	3 positive
	Prostate cancer	9	8 positive, 1 negative
***Normal human tissues***	Breast	2	1 negative, 1 positive
	Colon	2	2 negative
	Endometrium/myometrium	3	3 negative
	Eyeball (cross-section)	1	1 negative
	Heart	2	2 negative
	Kidney	3	3 negative ^¶^
	Adrenal gland	3	3 negative
	Liver	2	2 negative
	Lung	3	3 negative
	Lymph node	3	1 positive, 2 negative
	Meninges	3	3 negative
	Muscle (skeletal)	2	2 negative
	Thyroid	1	1 negative
	Pancreas	3	1 positive, 2 negative
	Prostate	3	1 positive, 2 negative
	Spleen	2	2 negative
	Stomach	2	2 negative
	Ovary	2	2 negative
	Skin	6	6 negative
	Testes	2	2 negative

*: samples from normal brains or from area of a glioblastoma multiforme patient’s brain diagnosed not to be involved in glioblastoma multiforme; ^‡^: Areas of glial cell reactivity show a few cells binding bClTx; ^§^: Metastatic tumors of unknown tissue origin; ^¶^: a few positive cells were observed.

Soroceanu *et al.* demonstrated that ^125^I-labelled CTX has both high and low affinity binding sites on glioma cells and is able to label cancer cells on biopsies of human patients affected with glioma [15]. They also showed that injection of ^131^I-CTX by IV route in SCID mice bearing human glioma tumor lead to specific peptide accumulation within the tumor. This study proves that CTX is able to label cancer cells *in situ* in the brain. A few years later, Lyons *et al.* showed that CTX binds to glioma cells as previously described but also to other tumors of the same neuroectodermal origin [16]. These additional studies, performed on over 200 tissue biopsies, include melanoma, small cell lung carcinoma, neuroblastoma, medulloblastoma, Ewing’s sarcoma and pheochromocytoma. These findings further extend the range of applications in which CTX may be used (Table 1). All these properties highlight the fact that CTX is a very attractive peptide for targeted cancer therapy or imaging. As a matter of fact, these properties were exploited by TransMolecular Incorporation that launched CTX for clinical trials under its trade name TM-601. After completion of clinical Phase II, the intellectual property rights on the molecule were acquired by Morphotek Incorporation, a US-based subsidiary of Eisa Corporation. In order to facilitate phenotyping and histological staining, a full line of CTX-labeled derivatives has been produced by TransMolecular Incorporation under the terms TM602, TM604, *etc*. We will give more details on the interest of TM-601 later in the review. So far, TM-601 is the only derivative of CTX for which human clinical studies are partially published.

This review article provides an overview of the research progress that has been made on CTX, namely on its mechanism of action and the development of CTX-derived compounds for the detection and treatment of glioma. A small part of our analysis will also be devoted to the discovery of chlorotoxin-like peptides of therapeutic potential.

## 4. Mode of Action of CTX: Looking for a Glioma-Specific Receptor

*Chloride channels*—Originally, CTX holds its name from its pharmacological effect on rat colonic epithelial cell chloride channels as described by Debin *et al.* in 1993 [12]. Small conductance Cl^−^ channels were shown to be potently blocked by CTX when the latter was applied towards the intracellular face of the channel [12,17]. After this initial characterization, CTX has been used as a general pharmacological tool to investigate the function of chloride channels. It is through this procedure that Ullrich *et al.* discovered the existence of specific CTX-sensitive glioma chloride currents in acute slices of human gliomas [18]. To further identify this receptor/ion channel, ^125^I-CTX binding to various malignant glioma cell lines (D54-MG, SK-1-MG, U87-MG, U105-MG, U251-MG and U373-MG) was investigated [15]. Using radioreceptor assays, the authors identified a 72 kDa band as the receptor of ^125^I-CTX. This molecular weight is in agreement with the molecular weight of CLC, a family of chloride channels [19]. Prolonged exposure to CTX results in cell internalization of this channel type [20]. What may seem as the most promising result is the fact that although gliomas come with an amazing degree of antigenic variability, they all seem to over-express this CTX-sensitive chloride current [21,22]. These channels are absent or in low abundance in healthy tissues or in tumors of non-glial origin [23]. Interestingly, expression of this channel type appears to be correlated with the histopathological tumor grade. High-grade tumors express more chloride channels than low tumor grades. The role of this channel type in glioma is still obscure but one suggestion is that it may facilitate the modifications in cell volume and shape that accompany glioma cell migration and healthy brain tissue invasion [24]. Indeed, Cl^−^ ions movement across the plasma membrane controls the cell volume changes. In turn, the change in glioma cell shape is required for cell invasion within the novel extracellular spaces created between healthy cells. This CLC chloride channel is therefore of potential importance for glioma malignancy. In this context, CTX would act by inhibiting Cl^−^ flux and limiting the extent of glioma cell shape alteration, thereby hampering the glioma tissue invasion potency. Other chloride channel inhibitors have been tested and also shown to inhibit glioma migration [8]. This model fits well with the reported anti-invasive effects of CTX on glioma cells and the inhibition of metastasis [5,8,20,21,25,26]. More information about the role of ion channels in glioma invasion can be found in several excellent review articles [8,25,26]. From the literature, it can be inferred that chloride channels constitute a marker of interest for the diagnosis of glioma and, because of their role in tumor growth, they can be used as a potential target for therapeutic approaches. In any case, the data point to the fact that the chloride channel may constitute a marker of interest for the diagnosis of glioma, and, because of its function in tumor growth, a potential target for therapeutic approaches. Nevertheless, the arguments in favor of a chloride channel as the actual target of CTX need to be balanced with more negative findings: (i) patch-clamp reports showing that, in spite of CTX-mediated chloride current block, the functional inhibition of the channel occurs with a lower affinity than expected from binding experiments (600 nM) [18]; and (ii) CTX has no effect on the proliferative rate of C6 glioma cells *in vitro* [24]. In fact, in their binding studies, Soroceanu *et al.* found two binding sites for ^125^I-CTX in glioma cell lines *in vitro*: a high affinity binding site with a K_d_ value of 4–9 nM and a low affinity one with a K_d_ in the 0.5–1 µM range. These findings may argue for the existence of more than one type of membrane receptors for CTX.

*Matrix metalloprotease MMP-2*—While searching for the molecular identity of the cell surface receptor of CTX, a 6His-tagged CTX analogue was designed and used to prepare an affinity column for mass spectrometry-mediated identification of CTX receptor from a solubilized human D54-MG glioma cell line. Surprisingly, the authors found that besides interacting with the ClC-3 chloride channel, CTX also brought along a complex of proteins that comprises membrane type 1 matrix metalloprotease MT1-MMP, matrix metalloprotease MMP-2 and tissue inhibitor of matrix metalloproteinase-2 TIMP-2 [5]. The matrix metalloprotease MMP-2 is expressed in glioma and other tumors but is not present in normal brain tissues. It is part of the larger family of metalloproteases that have been associated with the enzymatic degradation of the extracellular matrix (ECM). Excess matrix metalloprotease MMP-2 expression is therefore related to the easiness of the tissue invasion capability of glioma cells. All types of tumors reported to bind CTX were found to over-express the matrix metalloprotease MMP-2. This correlation between the expression level of MMP-2 and CTX binding supports the concept that the matrix metalloprotease MMP-2 may be part of the receptor complex of CTX. Within the protein complex interacting with CTX, the authors also identified the presence of αvβ3 integrin [20]. How integrins, matrix metalloproteases and chloride channels come together to interact with CTX remains a difficult issue to solve. Obviously, more research has to be done in terms of biochemistry and cell biology to define with which protein exactly CTX may interact to pull-down such a large protein complex. Considering the size of the peptide, it may appear unlikely that CTX can interact with all these protein partners simultaneously. It is, however, not uncommon that an animal toxin lacks selectivity and is capable of binding to several types of membrane receptors. Of course, the matrix metalloprotease MMP-2 also appears to be an interesting target for explaining the effects of CTX. The matrix metalloprotease MMP-2 is involved in ECM degradation, and the local enzymatic activity of this protein in the tumor environment should logically favor glioma cell division and migration. Interestingly, CTX has been observed to inhibit the enzymatic activity of the matrix metalloprotease MMP-2 and to promote endocytosis of this metalloprotease in glioma [5]. These two factors combined should reduce the extent of ECM degradation that can be sustained by the remaining matrix metalloprotease MMP-2 thereby providing another explanation of CTX-mediated inhibition of cell invasion. In any case, a compound that would prevent both ECM degradation and chloride channel-mediated cell shape alterations would be ideal. Conceptually, it is preferable to envision that Cl^−^ channels are associated with a complex of proteins formed by MMP-2, αvβ3 integrin, MT1-MMP and TIMP-2. CTX would not bind to individual receptors, but instead to this complex of proteins, and this binding would produce internalization of the entire protein complex, thereby leading to reduction of the activity of both the chloride channel and the MMP-2 [20]. This CTX-mediated internalization process would occur in caveolar rafts. In agreement with this concept, it was found that the effect of CTX on Cl^−^ current took over 15 min, a time lapse more compatible with receptor internalization than with ion channel blockade. Interestingly, the observation that CTX is also co-internalized during this process explains why the iodinated analogue of CTX is still visualized within tumors eight days after administration in clinical trials [27]. Arguably, the existence of such a protein complex that comprises both metalloproteinases and chloride channels makes sense for cancer cells because of the imperious need to degrade ECM and concomitantly alter cell morphology to facilitate the infiltration of tiny intercellular spaces. A study further reports that CTX, coupled to iron nanoparticles, inhibits the invasive nature of glioma cells *in vitro*, deactivates membrane-bound metalloproteinase 2, produces receptor-mediated endocytosis and inhibits cell volume changes [13].

*Annexin A2—*As if things were not complex enough, another research group now claims that the CTX receptor is annexin A2. This study was prompted by a contradictory report about MMP-2 as CTX receptor [14]. The observation was made that CTX also bound to a non-tumor cell type (proliferating human vascular endothelial cells) which also makes CTX a potent anti-angiogenic agent [28]. Based on this observation, TransMolecular Inc. reinvestigated the identity of the endothelial/glioma CTX receptor using affinity columns, cross-linking reagents and mass spectrometry. Annexin A2 was identified as the common receptor component that binds TM602, the biotinylated derivative of CTX, in all cell lines [29]. Annexin A2 is a calcium-dependent phospholipid binding protein present on the extracellular side of the plasma membrane of various tumor cells and endothelial cell types. It has many roles in cellular functions such as angiogenesis, apoptosis, cell migration, proliferation, invasion and cohesion. siRNA knockout of annexin A2 results in reduced binding of a technetium-99m-labelled-TM601 in cell lines expressing annexin A2. Interestingly, the same siRNA-mediated knockout decreases the *in vitro* migration of glioma cells [30]. In any case, these studies promote the idea that annexin A2 is an interesting therapeutic target in angiogenesis and tumor progression [31], although they dramatically complicate the landscape with regard to the identity of the real CTX receptor. In addition, while annexin A2 is involved in cell proliferation, migration and invasion, not much is known about how CTX binding to annexin A2 may hamper glioma invasion in the brain.

All these potential CTX receptors appear to be over-expressed in other tumors. This is the case of MMP-2 in breast, colon, skin, lung, prostate and ovary cancers, but also for annexin A2 in colorectal, pancreatic prostate and lung cancers, and hepatocellular and squamous cell carcinomas. This may explain why CTX also targets other cancer types [5,9,14,15,18,21,32] (Table 1). The multiplicity of claimed “true” receptors of CTX may seem disturbing at once unless one considers that they are all part of the same receptor complex. While MMP-2 and annexin-2 are all possible receptors at this stage, it is worth mentioning, however, that most animal toxins target ion channels. It may therefore come as a surprise that CTX may target (a) receptor(s) of a different nature than ion channels. Regardless of the nature of the true CTX receptor, the mechanism of CTX action on glioma needs refinement; most likely by a complete structure-function analysis that sorts out the structural determinants of CTX involved in binding on each one of these multiple potential receptors.

## 5. Targeted Imaging of Glioma by CTX

The first step in glioma treatment is surgical removal of the tumor mass if possible. Because of its great tendency to invade the normal brain tissue, it is very difficult for a surgeon to remove the entire tumor without leaving some cancerous cells located outside of the main tumor mass. Cancer cells that could not be removed by the surgeon will unavoidably cause the formation of new tumor mass(es) provoking the relapse of the patient. This is the reason why molecular tools for glioma detection and imaging are needed to provide efficient help to the surgeon and to evaluate the benefit of a different treatment than surgery. CTX is a small neurotoxin peptide that selectively binds to glioma cells. As it can be chemically synthesized and easily modified with appropriate imaging or therapeutic functions, several reports demonstrate that it can be used as a tool for glioma detection and imaging [14,27,33,34,35,36,37,38,39,40,41].

On the basis of this principle, three types of compounds have been designed. The first type is composed of CTX covalently coupled to a fluorescent indicator, Cy5.5. This tool should allow a surgeon to directly visualize cancer cells in real-time during tumor resection [14,33,34]. The second type of compound is composed of a Magnetic Resonance Imaging (MRI) contrast agent coupled to CTX as targeting ligand [27,35,36]. Finally, the third type of complex is composed of multifunctional nanoparticles, generally MRI contrast agents associated with optical imaging or therapeutic agents, together with CTX [37,38,39,40,41].

“Tumor paint” is a complex of CTX (as targeting agent) and Cy5.5, a near-infrared fluorescent (NIRF) molecule, first described by Veiseh *et al.* [14]. While CTX is able to specifically bind to glioma and related tumor cells, it cannot be detected in the absence of a convenient marker. Cy5.5 is an interesting marker because it emits photons in the near-IR spectrum. These types of photons have the particularity to be poorly absorbed by water and hemoglobin, making them compatible with intraoperative imaging. Bioconjugation of CTX to Cy5.5 was performed via NHS ester crosslinking. Cy5.5 NHS ester will react with CTX primary amines to form CTX-Cy5.5. The trouble with this approach is that there are four primary amines on CTX (three lysine residues (Lys^15^, Lys^23^ and Lys^27^) and the *N*-terminal amine group) that are all able to react with NHS ester Cy5.5, thus potentially yielding a mixture of mono-, di-, and tri-labeled peptides. Although it can be envisioned to use a mixture of labeled peptides to detect pathological tumor masses, it is technically challenging and costly to reproducibly preserve the exact ratio between these different labeled compounds [33]. A solution to this problem has been proposed by Akcan *et al.* [34]. In their report, they substituted the lysine residues at position 15 and 23 by alanine or arginine. These two lysine residues were targeted because they react minimally with Cy5.5 NHS esters. The conditions of labeling with Cy5.5 were chosen in such a way that the *N*-terminal primary amine was not labeled. According to this procedure, they obtained monolabeled CTX with Cy5.5 grafted onto Lys^27^ only. Cy5.5 labeling of a non-mutated, but cyclized CTX surprisingly also yielded a monolabeled peptide [34]. All these generated compounds (the original tumor paint CTX-Cy5.5 multi-labeled compound, the mono-labeled mutated CTX, and the cyclic labeled CTX) have kept their ability to target tumor cells. More studies have been conducted with the tumor paint CTX-Cy5.5 compound on both toxicity and biodistribution [14]. The bioconjugate is homogeneously distributed in mice upon IV injection. Renal accumulation could be observed, but not to abnormal levels since CTX itself is eliminated in the urine. No toxic effect was observed two weeks after exposure to CTX-Cy5.5 in mice. This compound will progress towards use in human clinical trials. It should ease the definition of glioma boundaries upon surgery and hopefully increase the mean survival time of the operated patients that may benefit of this technology.

MRI contrast agents are needed to visualize the precise glioma localization within the brain and determine the exact size of the tumors. Gadolinium ions (Gd(III)) are widely used MRI contrast agents when chelated in appropriate molecular cages (e.g., Gd(III)-diethylenetriaminepentaacetic acid (Gd-DTPA) from Magnevist and Gd(III)-N,N0,N00,N000-tetracarboxymethyl-1,4,7,10-tetraazacyclododecane (Gd-DOTA) [36]. They are stable and have low molecular weights. However, they are rapidly eliminated and do not possess intrinsic targeting abilities. In order to enhance the retention time of these Gd-based contrast agents in mice and add a targeting function to the contrast agent, Huang *et al.* created a dendrigraft poly-l-lysine (DGL) compound [36]. DGL is a l-lysine dendritic macromolecule that carries both Gd chelates and CTX. This compound allows a better uptake of the contrast agent by tumor cells and provides a targeting cancer cell property to the macromolecule. The retention time in the tumor was enhanced as expected and greater signal intensity was recorded. This compound shows no apparent toxicity, making it a good candidate as MRI contrast agent for glioma detection in the future.

Finally, nanoprobes have been used as MRI contrast agents as well. Contrary to Gd chelates, nanoprobes provide a better resolution of the edge of the tumor thanks to an improved cellular uptake and a slower clearance at the tumor site [35]. Nevertheless, they similarly do not possess any intrinsic tumor-targeting ability so that they seem to preferentially label the reticulo-endothelial system surrounding the tumor site. In order to overcome this problem, a new nanoprobe composed of an iron superoxide particle, coated with polyethylene glycol (PEG), and conjugated with CTX was designed [35]. The efficiency of this nanoprobe was evaluated *in vitro* and *in vivo* in mice. It shows a real improvement in tumor targeting efficiency compared to nanoprobes lacking CTX conjugation. These CTX-functionalized nanoprobes have no detectable toxic effects. The most interesting thing about these nanoprobes is that not all the PEG molecules are used to fix CTX. These nanoprobes can therefore be used as a conjugation platform not only to target and identify cancer cells, but also to fight cancer cells. It is with this view in mind that the same research group has designed improved nanoprobes [37,38]. This enhanced version of the nanoprobes [37] has better characteristics: (i) the probes pass the BBB or the BBTB and present reduced opsonisation properties; (ii) they are composed of biocompatible material and (iii) contain Cy5.5, an additional diagnostic component. This new nanoprobe offers combined MRI detectability and near-IR fluorescent detection. Because this compound has a demonstrated residency time exceeding five days in cancer cells, it would allow preoperative diagnostics, followed by intra-operating imaging during tumor resection and post-operative control. Moreover, with such a nanoprobe platform, there is additional room for further chemical functionalization in order to have other specific applications. Cy5.5 is not the only dye that has been used to create multifunctional superparamagnetic iron oxide nanoparticles. Fluorescein isothiocyanate (FITC) has also been successfully added to this type of nanoparticule [39]. Regarding the emission spectra of FITC, this nanoprobe can only be used *in vitro*. A good discrimination between glioma cells and healthy tissue has been described for this compound.

**Table 2:** Summary of various compound made with CTX.

Types of link	Cargos	Application	References
Covalently link to	*Iode*	Radiotherapy and Imaging	[3,9,15,28,32,35,40,42,43]
	^125^I,^131^I		
	*Fluorescent dyes*	Imaging and detection	[14,15,29]
	Cy5.5		
	Oregon green		
	*Drugs*	Therapeutic	[44,45]
	Platinium		
	Anticancer drugs		
	*Biotine*	Immunostaining detection	[15]
	*Nitric oxide*	Therapeutic adjuvant	[46]
Covalently link to a vehicle	*Nanoparticle*		
	Iron superoxides core	MRI contrast agent	[34]
	Multifunctional nanoprobes + Fluorescent dyes (Cy5.5, FITC, Alexa fluor)	MRI contrast agent and imaging agent	[13,36,37]
	Multifunctional nanoprobes + cDNA or siRNA	MRI contrast agent and therapeutic	[38,47]
	Multifunctional nanoprobes + Methotrexate	Therapeutic and MRI contrast agent	[39]
	PEI core + cDNA+ Fluorescent dyes	Therapeutic and imaging	[48,49]
	*Liposome* Doxorubicine loaded	Therapeutic	[50]
	*Dendrigraft poly Lysine* With Gadelinium	MRI contrast agent	[33]
	*Empty capsule of hepatitis B*	Future therapeutic vector	[51]

Besides imaging tumors, multifunctional nanoparticles have also been designed for cancer treatment (Table 2). Instead of a dye or other markers, drugs or siRNA, DNA is being used. Since the main interest of this type of compound is the therapeutic area, more details will be given in the next section. Developing dual imaging/therapeutic molecules based on CTX is of course not exclusively limited to multifunctional nanoprobes since ^131^I-TM601, a radiolabeled CTX, designed at first for radiotherapy of glioma, has shown an evident efficacy to visualize brain tumors by SPECT [27]. More details on this compound will be provided in the following subsection.

## 6. Chlorotoxin as Therapeutic Targeting Peptides

After glioma surgery has been performed, the next step to treat glioma is radiotherapy with or without chemotherapy. In this area, CTX is also used as a targeting peptide to precisely deliver a therapeutic agent or radionuclides (Table 2).

The first CTX compound developed was ^131^I-TM601. It entered clinical trial in 2002 in the USA. This compound is made of a synthetic version of CTX named TM-601 on which iodination was performed on Tyr^29^. ^131^I-TM601 is the subject of several publications [3,9,27,28,29,32,42,43,52], and many patents have been filled in to protect its use as a therapeutic drug. The Phase I study was conducted to determine the safety, biodistribution, tolerability and dosimetry of intracavitary injection of ^131^I-TM601 in adult patients affected with high grade glioma [42]. A single dose of intracavitary administration of 10 mCi of ^131^I-TM601 (0.25 to 1 mg of product) seems to be well tolerated. This result demonstrated the safety and the good tolerance of the patients to the product over the 180-day period observation. However, three of the eighteen patients developed adverse effects that were imputed to the mode of administration (through an Ommaya reservoir) rather than to ^131^I-TM601 itself. These adverse symptoms include high fever, chills, mild cerebral edema on computed tomography (CT), and infection of the tumor resection cavity. None of these secondary effects have impaired the continuation of the study. As expected, ^131^I-TM-601 accumulated in the tumor cavity margin. As such, radiation doses to normal tissue organs were insignificant. As no major toxicity and no death due to ^131^I-TM-601 have been reported, the FDA allowed the trial to go to Phase III [9,42,43]. An FDA approval has also been obtained to investigate the effect of TM601 on newly occurring glioma. According to TransMolecular Incorporation, TM601 is extremely stable, presents no immunogenicity and lacks toxicity in humans. This stability issue has been probed by the group of Olson that demonstrates that 70% of CTX remained intact after 24 h incubation in human serum at 37 °C, indicating the relative resistance of the peptide to peptidases [34]. The excretion route appears to be through the urinary tract. The benefit of the treatment with TM601 would be a two-fold increase in the patient’s lifespan, which is by itself a considerable advance considering the devastating rapid progression of the disease when diagnosed. Parallel to this trial, the same team has worked on an intravenous injection protocol of this product but this time with an imaging application in perspective (described earlier in our review) [27]. Interestingly, intracranial injection of ^131^I-CTX detects brain tumors by gamma-ray scintigram scans *in vivo*, but also labels the stomach, indicating that the molecular target of CTX is also expressed in this organ. So far, TM-601 is the only derivative of CTX for which human clinical studies are partially published.

Besides the clinical trial of ^131^I-TM-601, many other CTX applications have been described that may be potentially useful to treat glioma. A large proportion of them rely on the administration of therapeutic agents, cDNA or siRNA to block oncogene expression, thanks to the use of nanoparticles [40,41,47,48,49,53]. The use of two types of nanoparticles has been reported: (i) with a polymer core or (ii) an iron superoxide core (multifunctional nanoparticles). Polymers such as polyethylenimine (PEI) or poly(amidoamine) (PAMAM) have been used to administer cDNA to cancer cells [48,49]. These vectors have shown some success for internalization and transfection of cancer cells, providing researchers with a viable alternative to viral infection. Many differences underlie nanoparticles formed with polymer or iron cores, such as size, surface charge and surface composition, but the most interesting one resides in the fact that the iron core particle can also be used for MRI. In addition also, superparamagnetic iron oxide nanoparticles can be used for hyperthermia therapy of cancer cells. Thus, Veiseh and collaborators have designed two new nanoprobes based on the same platform (coated iron superoxide particles conjugated to CTX) that can be used as MRI contrast agent and deliver therapeutic agents (such as methotrexate and siRNA) to glioma cells [40,41]. Both compounds accumulate in glioma cells with a concomitant increase in methotrexate toxicity for nanoparticles coated with this anti-tumor agent and a better knock-down for the siRNA nanoprobe. Another team has worked on targeted gene therapy thanks to this multifunctional nanoparticle and successfully showed cell transfection with a plasmid coding for Green Fluorescent Protein. They prove that the use of this vector induces an improved selective uptake by cancer cells and thereby a better gene expression [47]. With these nanoprobes, the effect of the glioma treatment can be followed by MRI by inspecting nanoprobe accumulation in tumor cells. Other nanovectors have been described that contain CTX [13,51]. In these studies, while CTX is used for its vectoring properties, the complexes developed do not contain any cytotoxic agent. One first type of nanoparticle described has a rather classic composition since the iron nanoparticle is coated with PEG on which CTX and alexafluor 680 have been grafted [13]. A second type of study reports a particle with an original composition since it contains an empty capsule of hepatitis B virus on which a Fc antibody fragment was adsorbed [51]. It is on this antibody fragment that CTX has been conjugated. These types of particles show preferential binding onto glioma cells and the mere presence of CTX is sufficient to inhibit the invasion of these cancer cells. Further development of these compounds is needed to lead to new therapeutic vectors with CTX as targeting agent.

Nanoparticles are not the sole vectors useful to administer therapeutic agents to glioma. Xiang *et al.*, describes the administration of doxorubicin to cancer cells using liposomes labeled with CTX as a vehicle [50]. This vector leads to a better accumulation of doxorubicin in cancer cells and increases the toxic effect. Key results presented in this manuscript are of obvious interest since they circumvent the use of nanoparticles that have unknown effects in the human body following long-term administration.

Nanoparticle-based therapies are as a matter of fact less likely to enter clinical phase trials than more classical compounds. In other reports, making more direct use of the glioma targeting properties of CTX, the peptide has been covalently linked to active anti-tumor agents [45,46]. Graf *et al.* [45] describe the synthesis of a platinum (IV)-CTX conjugate. This compound was designed in order to create an equally effective analog of cisplatin. Cisplatin is one of the most widely used anticancer drugs that, unfortunately, has major negative side effects. This led researchers to find related analogues without the major drawbacks associated to cisplatin. Thus, in this report from Graf and collaborators, platinum (IV)-CTX conjugate has a cell toxicity closely related to the one of cisplatin with the add-on benefit of glioma targeting. Although the results look promising, additional studies have to be conducted to fully characterize this compound. Another recent report describes an innovative use of CTX [46]. This time, CTX is covalently bound to nitric oxide (NO) to form a diazeniumdiolate NO donor (the NO first reacts with free amines of CTX to form radicals which then react with another NO molecule to form the diazeniumdiolate compound) [54]. This compound is not meant to act as an anticancer drug but to induce chemo-sensitivity to the targeted cell. Thus, a subsequent administration of temozolomide or carmustine, two anti-tumor drugs, will improve the toxicity of these drugs towards glioma cells and, in any case, exceed the one observed in the absence of CTX-nitric oxide treatment. This compound is designed to locally enhance the toxic effect of known anticancer drugs. This article opens a new field of possibilities to treat cancer although the mechanism of action of NO has yet to be elucidated.

## 7. Chlorotoxin-Like Toxins

**Figure 2:**
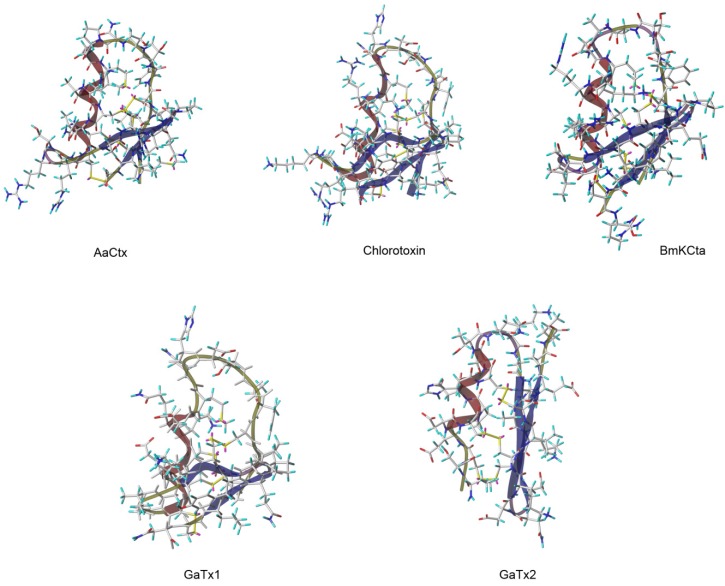
3D Representation of CTX and the other chlorotoxin like peptide. 3D structure were obtain from pdb files for Chlorotoxin (1CHL), from model of *SWISS-MODEL Repository* [65,66,67,68,69] for BmKCta, GaTX1 and GaTx2, and from model build by homology with CTX using @TOME V2 [64] and modeller for AaCtx.

Although many studies have attempted to analyze the mechanisms of action of CTX, structure-activity relationship studies are still curiously lacking. As CTX seems a promising lead compound to fight solid cancer, researchers have looked for the existence of other venomous toxins that may have the same potential as CTX. While quite a few peptides were identified, based on sequence homology and/or mode of action [55,56,57,58] (Table 3), only four of them share the CTX-reported activity on chloride channels. This is the case for AaCtx from the venom of *Androctonus australis* scorpion [59], BmKCTa from the venom of *Buthus martenzii* scorpion [60,61] and GaTx1 and GaTx2 both originating from the venom of *Leiurus quinquestriatus* scorpion [62,63] (Figure 2).

**Table 3:** Primary sequence alignments of chlorotoxin-like peptides. Alignments were performed by using @TOME V2 [64]. Percentage sequence of identity is given as compared to chlorotoxin by using @TOME V2 [64]. Disulfide Bridge pattern is given when known. Cysteine residues involved in disulfide bridges appear in blue in the table and are numbered in order of appearance.

Toxin	Primary sequence	Lenght	Identity	Disulfide bridge pattern	Species
**Chlorotoxin**	**M$C_{1}$MP$C_{2}$FTTDHQMARK$C_{3}$DD$C_{4}$$C_{5}$G-GK-GRGK$C_{6}$YGPQ$C_{7}$L$C_{8}$-R**	36 AA	100%	C_1_-C_4_,C_2_-C_6_,C_3_-C_7_,C_5_-C_8_	*Leiurus quinquestriatus quinquestriatus*
**I_1_**	**M$C_{1}$MP$C_{2}$FTTRPDMAQQ$C_{3}$RA$C_{4}$$C_{5}$K-GR-GK--$C_{6}$FGPQ$C_{7}$L$C_{8}$GYD-**	36 AA	71%	C_1_-C_4_,C_2_-C_6_,C_3_-C_7_,C_5_-C_8_	*Buthus eupeus*
**I_3_**	**M$C_{1}$MP$C_{2}$FTTDHQTARR$C_{3}$RD$C_{4}$$C_{5}$G-GR-GR-K$C_{6}$FG-Q$C_{7}$L$C_{8}$GYD-**	36 AA	82%	C_1_-C_4_,C_2_-C_6_,C_3_-C_7_,C_5_-C_8_	*Buthus eupeus*
**I_4_**	**M$C_{1}$MP$C_{2}$FTTDHNMAKK$C_{3}$RD$C_{4}$$C_{5}$G-GN---GK$C_{6}$FGPQ$C_{7}$L$C_{8}$NR**	35 AA	82%	C_1_-C_4_,C_2_-C_6_,C_3_-C_7_,C_5_-C_8_	*Buthus eupeus*
**I_5_**	**M$C_{1}$MP$C_{2}$FTTDPNMANK$C_{3}$RD$C_{4}$$C_{5}$G-GG-KK--$C_{6}$FGPQ$C_{7}$L$C_{8}$NR--**	35 AA	79%	C_1_-C_4_,C_2_-C_6_,C_3_-C_7_,C_5_-C_8_	*Buthus eupeus*
**I_5A_**	**M$C_{1}$MP$C_{2}$FTTDPNMAKK$C_{3}$RD$C_{4}$$C_{5}$G-GN-GK--$C_{6}$FGPQ$C_{7}$L$C_{8}$NR--**	35 AA	79%	C_1_-C_4_,C_2_-C_6_,C_3_-C_7_,C_5_-C_8_	*Buthus eupeus*
**Bs-8**	**R$C_{1}$KP$C_{2}$FTTDPQMSKK$C_{3}$AD$C_{4}$$C_{5}$G-GK-GKGK$C_{6}$YGPQ$C_{7}$L$C_{8}$----**	35 AA	80%	C_1_-C_4_,C_2_-C_6_,C_3_-C_7_,C_5_-C_8_	*Buthus sindicus*
**Lqh-8/6**	**R$C_{1}$SP$C_{2}$FTTDQQMTKK$C_{3}$YD$C_{4}$$C_{5}$G-GK-GKGK$C_{6}$YGPQ$C_{7}$I$C_{8}$APY-**	38 AA	72%	C_1_-C_4_,C_2_-C_6_,C_3_-C_7_,C_5_-C_8_	*Leiurus quinquestriatus hebraeus*
**PBITx1**	**R$C_{1}$KP$C_{2}$FTTDPQMSKK$C_{3}$AD$X_{4}$$C_{5}$G-GX--KX**	25 AA	64%		*Parabuthus schlechteri*
**Bs-14**	**-$C_{1}$GP$C_{2}$FTKDPETEKK$C_{3}$AT$C_{4}$$C_{5}$G-GI-GR--$C_{6}$FGPQ$C_{7}$L$C_{8}$NRGY**	36 AA	61%	C_1_-C_4_,C_2_-C_6_,C_3_-C_7_,C_5_-C_8_	*Buthus sindicus*
**Neurotoxin P2**	**-$C_{1}$GP$C_{2}$FTTDPYTESK$C_{3}$AT$C_{4}$$C_{5}$G-GR-GK--$C_{6}$VGPQ$C_{7}$L$C_{8}$NRI-**	35 AA	70%	C_1_-C_4_,C_2_-C_6_,C_3_-C_7_,C_5_-C_8_	*Androctonus mauretanicus mauretanicus*
**AaCtx**	**M$C_{1}$IP$C_{2}$FTTNPNMAAK$C_{3}$NA$C_{4}$$C_{5}$G-SRRGS--$C_{6}$RGPQ$C_{7}$I$C_{8}$----**	34 AA	61%	C_1_-C_4_,C_2_-C_6_,C_3_-C_7_,C_5_-C_8_	*Androctonus australis*
**GaTx1**	**-$C_{1}$GP$C_{2}$FTTDHQMEQK$C_{3}$AE$C_{4}$$C_{5}$G-GI-GK--$C_{6}$YGPQ$C_{7}$L$C_{8}$NR--**	34 AA	79%	C_1_-C_4_,C_2_-C_6_,C_3_-C_7_,C_5_-C_8_	*Leiurus quinquestriatus hebraeus*
**BmKCT**	**-$C_{1}$GP$C_{2}$FTTDANMARK$C_{3}$RE$C_{4}$$C_{5}$G-GI-GK--$C_{6}$FGPQ$C_{7}$L$C_{8}$NRI-**	35 AA	76%	C_1_-C_4_,C_2_-C_6_,C_3_-C_7_,C_5_-C_8_	*Buthus martensii*
**Bm12-b**	**-$C_{1}$GP$C_{2}$FTTDANMARK$C_{3}$RE$C_{4}$$C_{5}$G-GN-GK--$C_{6}$FGPQ$C_{7}$L$C_{8}$NRE-**	35 AA	76%	C_1_-C_4_,C_2_-C_6_,C_3_-C_7_,C_5_-C_8_	*Buthus martensii*
**Lepidopteran**	**R$C_{1}$GP$C_{2}$FTTDPQTQAK$C_{3}$SE$C_{4}$$C_{5}$G-RK-GG-V$C_{6}$KGPQ$C_{7}$I$C_{8}$GIQ-**	37 AA	63%	C_1_-C_4_,C_2_-C_6_,C_3_-C_7_,C_5_-C_8_	*Buthus tamulus*
**BtITx3**	**R$C_{1}$PP$C_{2}$FTTNPNMEAD$C_{3}$RK$C_{4}$$C_{5}$G-GR--GY-$C_{6}$ASYQ$C_{7}$I$C_{8}$PG--**	35 AA	53%	C_1_-C_4_,C_2_-C_6_,C_3_-C_7_,C_5_-C_8_	*Buthus tamulus*
**GaTx2**	**--VS$C_{1}$--------ED$C_{2}$PDH$C_{3}$STQK-ARAK$C_{4}$DNDK$C_{5}$V$C_{6}$-EPI**	29 AA	38%	C_1_-C_4_,C_2_-C_5_,C_3_-C_6_	*Leiurus quinquestriatus hebraeus*

AaCTx is a small peptide composed of 34 amino acid residues. It is present in a low concentration (0.05%) in the venom of *Androctonus australis.* It also contains eight cysteine residues, all engaged in disulfide bridges with a pairing pattern that is identical to the one observed in CTX. This toxin has 61% identity (75% sequence homology) with CTX. Overall, the amino acid sequence of AaCtx differs from that of CTX by 12 amino acids, which induce a change in the net charge of the peptide of +4 for AaCtx *vs.* +3 for CTX. Rjeibi *et al.*, characterized this peptide more precisely [59]. They investigated a potential neurotoxic effect in mice. Intra-cerebroventricular injection of 1 µg AaCtx produces no toxic symptoms. With regard of the high dose used in this study, these authors considered AaCtx as non-toxic for mammals. They also considered the effect of AaCtx on glioma cell migration and tissue invasion. They found a dose-dependent inhibition of migration and invasion with an IC_50_ of 125 µM and 10 µM, respectively. CTX acts on both processes at a lower concentration (600 nM) [8], indicating that AaCtx has a lower efficiency than CTX. A rapid screening for a mechanism of action of the peptide on migration and invasion of glioma cells did not reveal any evident effect on the extracellular matrix proteins (fibronectin, fibrinogen, vitronectin, laminin and collagen type IV). The authors made the assumption that AaCTx blocking effect on invasion and migration was due to a blockage of chloride channels. They linked the difference in net charges with CTX and also the substitution of acidic amino acid by neutral ones to the lower activity of AaCtx. Obviously, and again, only complete structure-activity relationship studies will be able to confirm the relevance of chloride channels as therapeutic target of these scorpion toxins.

BmKCTa is a component of the Chinese scorpion *Buthus martenzii karsh*. The venom of this scorpion was shown to induce glioma cell apoptosis and inhibit glioma tumor growth *in vivo*. Many bioactive compounds have been identified in this venom and there are still more to come [70,71]. The first description of BmKCTa dates back to the characterization of the cDNA sequence encoding it [60]. This peptide is composed of 36 amino acids and shares a high 68% sequence identity with CTX. It was first expressed in a bacterial system, and acute toxicity assays in mice were performed in 2005. An LD_50_ value of 4.3 mg/kg was determined in mice [61]. BmKCTa is, without any doubt, the most investigated CTX-like peptide. Comparative toxicity studies on glioma cells and astrocytes, patch-clamp experiments, and histological analyzes were conducted to further define the properties of this peptide and assess its potential as a therapeutic agent against human gliomas. BmKCTa inhibits SHG-44 glioma cell growth in a dose-dependent manner with an IC_50_ of 0.28 µM while the IC_50_ for normal astrocytes is 8 µM. This result indicates the extent of cell specificity in the toxicity of BmKCTa for glioma cells. In parallel, whole-cell patch-clamp recording shows the inhibiting effect of BmKCTa on chloride currents in SHG-44 cells. The histological analysis of BmKCTa in mice demonstrates that brain, skeletal and cardiac muscles are all target organs [72]. Similarly to CTX, BmKCTa is of potential interest as therapeutic agent against glioma and has also been found to bind to MMP-2 [73,74]. Both of these similitudes have led to the development of a compound based on BmKCTa to treat and image glioma [75]. An international patent has been filled in to use BmKCTa as an anti-tumor translocation peptide [76].

GaTx1 and GaTx2 are two toxins extracted from the venom of *the Leiurus quinquestriatus hebraeus* scorpion. GaTx1 is a peptide of 34 amino acid residues (79% homology sequence with CTX), which acts on cystic fibrosis transmembrane conductance regulator (CFTR). This receptor is a member of the ABC family but also possesses intrinsic Cl^−^ channel activity. It is known for its implication in cystic fibrosis. GaTx1 is a specific inhibitor of the CFTR channel that acts through reversible binding on a receptor site localized on the cytoplasmic side. It provides researchers with an interesting molecular tool for quantitatively dissecting the functional role of CFTR [62]. GaTx2 is a peptide of 29 amino acids that has poor sequence homology with CTX (38%). It is, however, also described to act on another Cl^−^ channel (ClC-2) than CTX (ClC-3) [63]. This channel is a member of the same family of chloride channels as ClC-3, and is also up-regulated on the glioma cell surface. Its physiological role, however, remains undefined although it can reasonably be assumed to play a similar role as ClC-3 in glioma cell invasion and migration [26]. GaTx2 inhibits ClC-2 by slowing its activation, and the resulting inhibition is voltage-dependent. Interestingly, the GaTx2 sequence was described earlier in 1997 under the name of leiuropeptide II without undergoing extensive characterization [77]. Nevertheless, GaTx2 can be used as a pharmacological tool to help determine the role and localization of ClC-2 channel in cells. It may serve as a scaffold/vector to design drugs that target ClC-2 channels. It is worth mentioning that because ClC-2 channels are also highly expressed in glioma cells [18] indicating that this toxin should also possess glioma-targeting properties. This remains to be tested of course.

Apart from these four toxins, two other animal toxins are worth mentioning here. First, Lqh-8/6 isolated from the venom of *Leiurus quinquestriatus hebraeus* scorpion, and Bs14 isolated from the venom of *Buthus sindicus* scorpion. Both are small peptides of 38 and 36 amino acids, respectively. They both share a high sequence and structure similarity with CTX (72% and 61% sequence identity, respectively). Each of them has eight cysteine residues in its sequence leading to the same compact folded structure and disulfide pairing pattern found in CTX. All of this evidence suggests that chloride channels should be their natural targets. However, at the current time, only the structures of these toxins have been studied, leaving us with the hope that new CTX toxin analogues, also possessing glioma targeting properties, will soon be discovered.

## 8. Conclusions

Since its discovery, CTX has been established as a promising tool to foster research on glioma. Although its mechanism of action is far from being fully elucidated, many applications have emerged to use its cancer-targeting properties. So far CTX has been linked to nanoparticles, radioisotopes and fluorescent molecules. In addition, it has been used to enhance the efficacy of already existing anti-tumor molecules and conjugated to active drugs to create new, more effective ones. One compound (^131^I-TM601) is already in Phase III of clinical trial. Other positive clinical perspectives for CTX or CTX-like toxins, include diagnosis by tumor paint using the reputed Cy5.5 dye or still more infrared-friendly dyes, tumor treatment with ^131^I and possibly the grafting onto CTX of other cytotoxic agents regularly used in clinics for cancer treatment such as cisplatin-like compounds, anthracyclines, or reactive oxygen species. All of this effervescence demonstrates the keen interest surrounding this toxin. CTX is one of the first target compounds that is not an antibody or an antibody fragment. Natural selection has made CTX very adapted to insects and other invertebrates but with only a small link to humans. This has led to a small, compact, highly diffusible peptide that can cross the BBB or the BBTB with so far no evident signs of toxicity for normal human cells. The issue of how well CTX crosses the BBB or the BBTB remains to be seriously investigated, as both proper cancer cell labeling and treatment will also require the targeting of cancer cells at the invasion front or at secondary tumor sites where the BBB may be well preserved. Obviously, CTX is not perfect. While it can inhibit tumor invasion, CTX cannot kill cancer cells on its own, obliging scientists to perform a complicated and costly work in designing and chemical grafting of compounds that provide this desired toxic property to the new molecules. Another point that needs to be addressed in the future is the absence of detailed structure activity relationship studies between CTX and its numerous potential targets. The absence of relevant information complicates the definitive identification of the real cell surface marker of glioma cells and will limit for some time the unambiguous identification of CTX-like toxins and their resulting characterization in the frame of glioma detection, diagnosis, imaging and treatment. Regarding the promising results obtained so far with CTX, there is no doubt, however, that more studies will be initiated in a few years to finalize the identification of the CTX target. Another source of hope consists of chlorotoxin-like toxins. It looks as if they are a promising reservoir of biologically active peptides with characteristics similar to or resembling those of CTX. In addition, since the vast majority of scorpion venoms remain to be characterized, there are still many more new compounds that will soon emerge with the same therapeutic promises, possibly targeting other ion channels of interest, such as Ca^2+^-activated small conductance K^+^ channels. Animal toxins may soon become an interesting source of promising therapeutic substances like plants have been before. CTX is on its way to definitively establish itself as a proof of concept of this emerging theory.
